# Hypokalemic Periodic Paralysis Secondary to Medullary Sponge Kidney Complicated With Renal Tubular Acidosis

**DOI:** 10.7759/cureus.30160

**Published:** 2022-10-10

**Authors:** Jia Li

**Affiliations:** 1 Diabetes and Endocrinology, Hanchuan People’s Hospital, Xiaogan, CHN; 2 Diabetes and Endocrinology, Wuhan University of Science and Technology, Wuhan, CHN

**Keywords:** secondary hyperparathyroidism, urinary ph, urinary potassium, non-gap, muscle weakness, numbness in the limbs, hyperchloremia, medullary sponge kidney, renal tubular acidosis, hypokalemic periodic paralysis

## Abstract

Hypokalemic periodic paralysis has a high risk of life-threatening dysrhythmias. Hyperchloremic acidosis with hypokalemia is a dangerous condition. There are several causes of hypokalemia, in addition to common diseases, such as hyperthyroidism, hyperaldosteronism, and Cushing’s syndrome; the other rare diseases include renal tubular acidosis (RTA), Bartter’s syndrome, and Gitelman’s syndrome. We present an unusual case of hypokalemic periodic paralysis, which was caused by a medullary sponge kidney with distal RTA. The patient had no significant medical history and was not taking any conventional drugs. Investigations demonstrated a combination of hypokalemia, hyperchloremia, metabolic acidosis with a normal anion gap, relatively raised urinary pH, and decreased phosphate level. Results suggested a diagnosis of RTA with secondary hyperparathyroidism. After potassium citrate replacement and correction of acidosis, the patient’s condition was in remission. This case highlights the rare etiology of hypokalemia and the need to actively search for the pathogenesis of unexplained hypokalemia to avoid delaying the condition.

## Introduction

Medullary sponge kidney (MSK) is a congenital renal cystic lesion with insidious onset. Most cases are found accidentally in adults, and renal tubular acidosis (RTA) is often the first manifestation [1]. Distal renal tubular acidosis (dRTA) is a rare condition characterized by abnormal urine acidification in the connecting tubule and the collecting duct. It is caused by a transport defect involved in the secretion of hydrogen ions, resulting in significant acid-base abnormalities, such as hyperchloremia, hypokalemia, and metabolic acidosis. It may be a fatal condition due to severe acute-subacute multiple electrolytes abnormality including hypokalemia [2]. dRTA is associated with multiple factors such as drugs, infection, and autoimmune diseases, and is more common in children [3]. Here, we describe the case of a middle-aged woman with muscle weakness and hypokalemic periodic paralysis due to severe hypokalemia, which was secondary to MSK and dRTA.

## Case presentation

A 45-year-old female teacher was admitted to the hospital with recurrent limb weakness and numbness and an inability to walk for 10 months. There was no history of smoking, alcohol use, or illicit drug use. Additionally, there was no history of genetic and infectious diseases. She started to develop numbness in the hands and weakness in the limbs 10 months ago, suddenly collapsed, and could not stand intermittently. These were combined with symptoms such as dry mouth, polydipsia, and polyuria, but there was no associated history of headache, nausea, vomiting, fever, chest pain, or dyspnea. On presentation to the Department of Neurology, hypokalemia was noted, for which potassium chloride was orally administered, and the above symptoms were relieved.

Eight months ago, she had presented to the Emergency Department with limb weakness over the preceding 10 days. An electrolyte test suggested severe hypokalemia, and she was admitted to the Endocrinology Department for hospitalization. Physical examination indicated normal temperature, clear spirit and consciousness, heart and respiratory rate were normal, oxygen saturation was normal, and the blood pressure (147/99 mmHg) was slightly higher. She had bilateral upper limb weakness (right power of 1/5, left power of 2/5) and lower limb weakness (power of 1/5), and there was a slight decrease in muscle tone. Moreover, the tendon reflex was positive. At the same time, there was a prickling sensation in the limbs. Blood gas analysis demonstrated hypokalemia with lower blood pH. The blood glucose, routine blood analysis, thyroid function, and liver and kidney function were normal. Electrocardiogram demonstrated sinus bradycardia and T-wave depression. Total abdominal computed tomography (CT) showed scattered stones in both kidneys. Ultrasound of the abdomen was normal, and CT of the head and chest showed no obvious abnormalities. Aldosterone was two times higher than normal, but the renin and aldosterone-to-renin ratio (ARR) were normal, suggesting increased secondary aldosterone. Cortisol level and rhythms were normal, and autoimmune antibodies were negative. After potassium supplementation, her limb muscle strength returned to normal. She was recommended to go to a high-level hospital for further examination to seek the cause of hypokalemia.

In a few weeks, her dynamic salivary gland imaging was performed which revealed normal findings in the referral hospital. Autoimmune antibodies and complements, C-reactive protein (CRP), renal function, and erythrocyte sedimentation rate were normal. The electrolyte test indicated hypokalemia, hyperchloremia, and hypophosphatemia. Blood gas analysis indicated that the pH was lower than normal. A routine urine analysis indicated that the urinary pH was higher than normal. Renin, aldosterone, and ARR were normal, which led to the exclusion of primary hyperaldosteronism and Sjogren’s syndrome.

**Figure 1:**
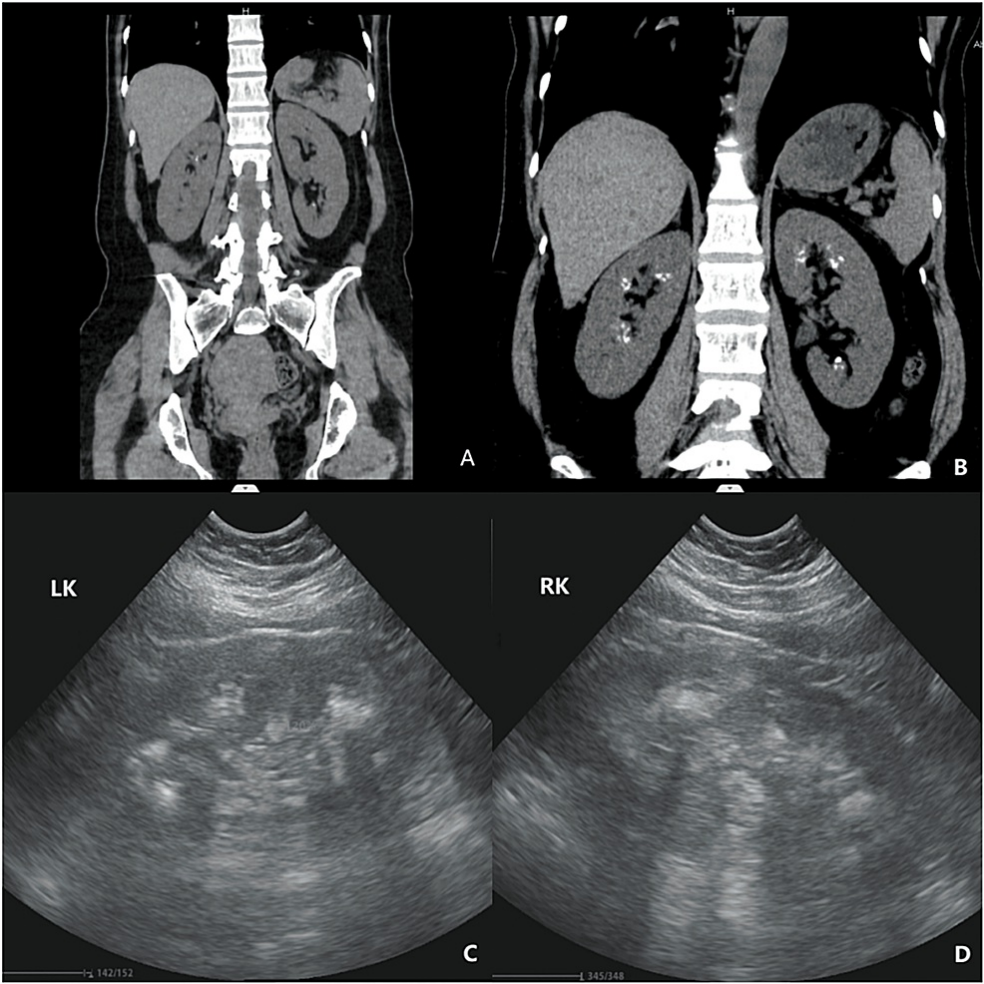
CT scan and color Doppler ultrasonography of both kidneys. (A) CT showing scattered stones in both kidneys. (B) CT showing high-density shadows in the renal medulla, with bouquet-like changes, indicating spongy kidneys. (C, D) Ultrasonography showing strong echoes, showing a fan-shaped arrangement, indicating spongy kidneys.

A few days later, she was admitted to the Urology Department because of pain in the right kidney area. Ultrasound showed multiple stones and calcification foci in the parenchyma of both kidneys. One week later, she was hospitalized again in our department for weakness and numbness in the extremities, indicating bilateral upper limb weakness (power of 4/5) and lower limb weakness (power of 3/5). Blood gas analysis and electrolyte test findings are shown in Table 1. Inflammatory biomarkers were normal, and an infection was ruled out. Bone mineral density was normal. Urinary pH and 24-hour urine potassium (NK) are shown in Table 2. An abdominal CT scan suggested multiple calculi in both kidneys and indicated MSK (Figure 1B). Renal and adrenal ultrasonography suggested increased echo in the pyramid of bilateral kidneys (similar to sponge kidney) (Figures 1C, 1D).

**Table 1:** Laboratory tests including serum chemistry and arterial blood gas analysis.

Blood chemistry	Result	Reference range
Sodium (mmol/L)	139.5	135–146
Potassium (mmol/L)	3.0	3.5–5.2
Chloride (mmol/L)	121.1	96–110
Bicarbonate (mmol/L)	13	23–30
Anion gap	8.4	8–16
Creatinine (mmol/L)	67	44–106
Urea (mmol/L)	4.07	2.86–8.20
Serum-corrected calcium (mmol/L)	2.03	2.11–2.52
Phosphate (mmol/L)	0.38	0.85–1.51
Magnesium (mmol/L)	1.32	0.75–1.02
Glucose (mmol/L)	5.6	4.0–7.0
Thyroid-stimulating hormone (mU/L)	1.12	0.49–4.91
Parathormone (pg/mL）	207	6–80
8 am cortisol (µg/dL)	13.10	5–25
4 pm cortisol (µg/dL)	5.45	3–13
Renin (µIU/mL)	40.1	4.4–46.1
Aldosterone (pg/mL)	242.0	<353.0
Total protein (g/L)	64.1	60–80
Albumin (g/L)	38.7	35–50
Globulin (g/L)	25.4	20–35
Arterial pH	7.244	7.35–7.45
Arterial pCO_2_ (mmHg)	28.7	35–45
Arterial pO_2_ (mmHg)	96.7	83–108
Arterial bicarbonate (mmol/L)	13.9	23–30
Arterial lactate (mmol/L)	1.2	0.2–1.8

**Table 2:** Urine routine test

Urine test	Result	Expected range
Potassium (mmol/L)	17.7	<15.0
Protein (g/L)	Negative	
Ketone body	Negative	
Glucose (mg/dL)	Negative	
Leukocyte (pcs/µL)	0	0–28
Erythrocyte (pcs/µL)	3	0-17
pH	7.5	<5.5
Specific gravity	1.010	1.005–1.030

**Figure 2:**
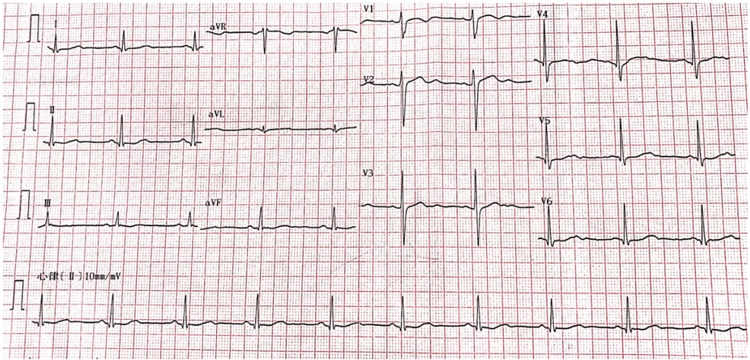
Electrocardiogram performed during admission. Electrocardiogram showing sinus bradycardia and low and flat T-waves.

The patient redemonstrated hypokalemia, and repeated electrolyte examination suggested hypokalemia combined with hyperchloremia and hypophosphatemia, decreased blood bicarbonate and pH, and increased urine potassium (>15 mmol/L), indicating increased renal potassium excretion. Moreover, urinary pH was higher than normal (>5.5), there was no anion gap, and there were secondary T-wave changes due to hypokalemia in ECG (Figure 2). CT and ultrasound suggested bilateral sponge kidneys. Glucose, ketone bodies, and protein were negative (Table 2), and liver and kidney function, thyroid hormones, renin, aldosterone, and cortisol were normal (Table 1). Autoimmune antibodies and complements were negative (Table 3), and the salivary gland dynamic imaging was normal, which excluded primary aldosteronism and autoimmune diseases.

**Table 3:** Protein electrophoresis and autoimmune workup.

Test	Result	Reference range
Immunoglobulin G (g/L)	9.5	8.0–16.0
Immunoglobulin A (g/L)	1.59	0.7–3.3
Immunoglobulin M (g/L)	0.93	0.5–2.2
Complement factor 3 (g/L)	0.99	0.8–1.6
Complement factor 4 (g/L)	0.26	0.2–0.4
Other autoimmune antibodies including anti-double-stranded DNA (anti-dsDNA), anti-nucleosome, anti-histone antibody, anti-Smith (Anti-Sm), anti-proliferating cell nuclear antigen (anti-PCNA), anti-ribosomal-P, anti-Sjögren’s syndrome A (anti-SSA) (Ro60), anti-Sjögren’s syndrome A (anti-SSA) (Ro52), anti-Sjögren’s syndrome B (anti-SSB), anti-centromere, anti-scleroderma antibody (anti-Scl-70), anti-ribonucleic protein (anti-RNP), anti-Jo-1, anti-PM/Scl, anti-Mi-2	Negative	
Protein electrophoresis	Normal	

Finally, the following conclusions were reached: (1) MSK with RTA, (2) hypokalemic periodic paralysis, and (3) secondary hyperparathyroidism. Due to persistent hypokalemia and metabolic acidosis, the patient was treated with sodium bicarbonate and potassium. After five days of intensive therapy, she was discharged from the hospital. Her condition was stable, the blood potassium was within the normal range during regular follow-up, and the symptoms were significantly improved.

## Discussion

MSK is a congenital cystic lesion of the renal medulla, with an incidence of 1/20,000 to 1/5,000 [4]. It is characterized by dilatation of the distal collecting duct and is mainly diagnosed by renal ultrasound and CT. MSK is mostly sporadic, and a few cases have a genetic tendency showing autosomal dominant or recessive inheritance. MSK is related to the dysfunction of renal tubule concentration which leads to RTA. Most of them are bilateral renal lesions located in the renal medulla and papilla, often involving multiple cones. Some patients are diagnosed in middle age due to renal colic, kidney stones, urinary tract infection, and hematuria [5]. Ultrasonography shows a strong echo in the kidney, consistent with the distribution of the renal pyramid, arranged in clusters and fan-shaped, around the collecting duct. CT scan shows plaque-like and high-density shadows in the renal medulla, which could be single or multiple, with bouquet-like changes, without hydronephrosis.

dRTA is caused by the dysfunction of distal renal tubule secretion and the reduction of luminal hydrogen ions, resulting in the replacement of hydrogen ions with potassium ions, leading to the exchange of potassium ions with sodium ions. A large amount of potassium ions is excreted from urine, and urine cannot be acidified normally, thus causing hypokalemia and non-gap hyperchloremic metabolic acidosis [6]. Studies have shown that this disease has genetic heterogeneity and various gene variants are associated with dRTA [7], with typical representatives including *SLC4A1*, *ATP6V1B1*, and *ATP6V0A4 *genes [8]. Hypokalemia caused by renal potassium loss leads to prominent manifestations, such as muscle weakness and paralysis. Patients may also have headache, fatigue, nausea, and vomiting, and severe acidosis may lead to malignant arrhythmias, coma, and even cardiac arrest. In children, due to chronic metabolic acidosis, developmental retardation and bone loss occur often, leading to rickets and osteomalacia. Chronic renal calcification, increased urinary pH, and hypercalciuria can progress to chronic kidney disease.

RTA and hypokalemia caused by MSK are rare in the clinic. In addition to anatomical abnormalities of renal collecting duct development, MSK can be accompanied by decreased urine concentration and abnormal renal acidification, leading to RTA. The reason is that the ability of distal convoluted tubules and collecting ducts to secrete hydrogen decreases or secreted hydrogen returns to blood, resulting in hydrogen retention and acidosis. Due to the insidious onset of MSK, RTA can be the first manifestation. Some studies have shown that about 50% of MSK patients have dRTA, and dysfunction of hydrogen secretion in distal convoluted tubules leads to the decrease of urine acidification in nearly 80% of MSK patients. Long-term renal excretion of water, sodium, potassium, calcium, and phosphorus increases, with the most prominent manifestations being hypokalemia and hypocalcemia. MSK causes the disorder of calcium metabolism, and long-term urinary calcium increase leads to hypocalcemia, resulting in secondary hyperparathyroidism. This patient had low blood calcium and phosphorus, with higher parathyroid hormone, which led to the consideration of secondary hyperparathyroidism, with an unclear mechanism. RTA can also lead to a high level of urinary calcium; hence, her hyperparathyroidism could be the result of a combination of the kidney and endocrine systems.

dRTA can be divided into hereditary, secondary, and idiopathic types, and the majority (about 89.91%) of adults are secondary types. The diagnosis of dRTA includes acidosis, normal anion gap, hyperchloremia and hypokalemia, and urinary pH of more than 5.5. This disease presents a normal anion gap, which reflects a decrease in urinary ammonium excretion and helps to distinguish metabolic acidosis from other diseases. The diagnosis of dRTA was based on 24-hour urinary potassium excretion, nephrocalcinosis confirmed by renal ultrasound or CT, and failure to reduce urinary pH below 5.5 after ammonium chloride loading or diuresis. The deficiency of this case is the failure to perform ammonium chloride testing. Secondary RTA is more common in women and often misdiagnosed clinically. This patient had previously been misdiagnosed with secondary aldosteronism. She had no history of severe illness, recurrent hypokalemia, and months of fatigue symptoms were ignored. Therefore, genetic testing is required if no underlying cause of RTA has been identified. Treatment of dRTA includes the replacement of potassium citrate and correction of metabolic acidosis, as well as prevention and evaluation of potential complications. For chronic dRTA, except potassium citrate and sodium bicarbonate is recommended which could correct acidosis. Potassium citrate can not only correct hypokalemia but also prevent the formation of kidney stones [9].

Several studies have confirmed that chronic hypokalemia can lead to renal cysts and interstitial fibrosis, followed by urinary concentration dysfunction. The mechanism of renal cystic degeneration caused by chronic hypokalemia is not completely clear, and it is speculated that it may be related to the growth and proliferation of epithelial cells in the cyst stimulated by hypokalemia [10]. MSK is one of the most classic cystic lesions, which is associated with hypokalemia, RTA, and nephrocalcinosis [11]. Studies have shown that patients with RTA must be complicated with renal cysts, and most of them are multiple in the kidneys [12], which indicates that under the condition of RTA, the chance of renal cysts caused by hypokalemia increases. In this case, a CT scan of both kidneys was performed eight months ago (Figure 2A), and sponge kidneys were not found at that time, perhaps because of the early stage of the disease or other reasons. Therefore, the stimulation of repeated hypokalemia is considered to be the cause of the development of sponge kidneys, and there may be a mutually reinforcing effect between them.

Hypokalemic periodic paralysis is a neuromuscular disorder associated with defective muscle ion channels and characterized by attacks of muscle weakness. The majority of cases are inherited in an autosomal dominant pattern. The consensus diagnostic criteria include the following aspects: first, two or more episodes of muscle weakness with serum potassium <3.5 mmol/L or one relative had a similar attack. Second, three or more of the following features should be present: onset in the first or second decade, onset time longer than two hours, the presence of triggers (previous carbohydrate-rich meal, onset during rest after exercise, stress), symptomatic relief with potassium intake, a family history of skeletal calcium or sodium channel mutation, and positive long exercise tests. Finally, other causes of hypokalemia (renal and adrenal disease, thyroid dysfunction, drug abuse) were excluded. In this case, the patient had repeated muscle weakness and delayed paralysis of limbs, mainly in the lower limbs, without any symptoms of neurological damage such as disturbance of consciousness or paresthesia. Moreover, the serum potassium was significantly lower than 3.5 mmol/L during the attack, and the electrocardiogram showed hypokalemic changes. The symptoms were relieved rapidly after potassium supplementation; therefore, hypokalemic periodic paralysis was considered. Unfortunately, electromyography and genetic tests were not performed for this patient, the latter may be used to differentiate between primary hypokalemic periodic paralysis and the other possible causes. Although it was presumed to be caused by RTA in this case, we will track these results if possible.

## Conclusions

The patient was a middle-aged woman with recurrent weakness of the limbs, which suggested hypokalemia. After potassium supplementation, the muscle strength of the limbs returned to normal. The patient had no other diseases commonly associated with hypokalemic periodic paralysis, and previous biochemical tests had repeatedly suggested hypokalemia with acidosis, increased blood chloride level, decreased blood phosphate level, and urinary pH >5.5 indicated classical RTA (type I) with secondary hyperparathyroidism. Combined with renal ultrasound and CT, RTA caused by MSK was diagnosed, which could lead to hypokalemic periodic paralysis. At present, there is no effective way to prevent and treat MSK, and the main treatment is to treat the complications. For hypokalemia secondary to RTA, potassium citrate is the best potassium supplement; in addition, anti-infection, correction of acid-base balance and electrolyte disorder, and intervention of stone production and discharge are also the key to the therapy. This case suggests that for patients with long-term clinical manifestations of hypokalemia, the etiology should be actively sought, and early diagnosis, early treatment, and regular follow-up are crucial to avoid chronic hypokalemia leading to renal cysts, which may aggravate or promote the deterioration of renal function, as well as unexpected complications.
